# Cancer immunotherapy nursing care from 2004 to 2023: a bibliometric analysis

**DOI:** 10.3389/fonc.2025.1508029

**Published:** 2025-06-16

**Authors:** Linjuan Li, Ruyu Ge, Tingting Wu, Hongyan Du, Xuanwei Zhang, Shiqi Tao, Linjun Zhong, Mingxia Li, Yan Fu, Yang Zhang

**Affiliations:** ^1^ Thoracic Oncology Ward, Cancer Center, West China Hospital, Sichuan University/West China School of Nursing, Sichuan University, Chengdu, China; ^2^ Obstetrics and Gynaecology, Jintang Maternal and Child Health Hospital, Chengdu, China; ^3^ Division of Thoracic Tumor Multimodality Treatment, Cancer Center, West China Hospital, Sichuan University, Chengdu, China; ^4^ Chinese Evidence-Based Medicine Center, West China Hospital, Sichuan University/Department of Medical Publishers, West China Hospital, Sichuan University, Chengdu, China

**Keywords:** bibliometric analysis, cancer immunotherapy nursing care, CiteSpace, VOSviewer, visualization analysis

## Abstract

**Objective:**

Cancer immunotherapy has become a leading treatment, but research on nursing care specific to this field remains underexplored. This study aims to analyze global trends in cancer immunotherapy nursing care from 2004 to 2023, focusing on key aspects such as patient education, symptom management, immune-related adverse event monitoring, and psychological support.

**Methods:**

The objective of this study was to explore global research trends in cancer immunotherapy nursing care from 2004 to 2023 through bibliometric analysis. A total of 4526 peer-reviewed articles from the Web of Science Core Collection (WoSCC) were selected based on the following criteria: 1) articles related to cancer immunotherapy nursing care, 2) publications written in English, and 3) publications from January 1, 2004 to December 31, 2023. Articles were excluded if they did not meet these criteria or were not directly related to immunotherapy nursing care. The analysis included annual publication trends, country and institution productivity, co-citation analysis, keyword analysis, and burst detection analysis. Tools such as GraphPad, CiteSpace, and VOSviewer were used to visualize the data and identify influential authors, journals, and research topics.

**Results:**

A total of 4526 publications were included. The publication trend showed a consistent annual increase. The United States was identified as the most prolific country, with the University of Texas System being the most productive institution. Cancers published the greatest number of articles in this field. Powles Thomas and Martin Reck were the authors who authored or co-authored the largest number of research papers. The research hotspots included melanoma, non-small cell lung cancer, cytokine release syndrome, and immune-related adverse events. Moving forward, future research could explore enhancing nursing interventions for immune-related side effects and developing standardized care protocols to improve patient outcomes.

**Conclusion:**

This study provides valuable insights into the research trends and developments in cancer immunotherapy nursing care over the past two decades. The findings also suggest potential practical applications in clinical nursing practice, particularly in the management of immune-related adverse events and patient education during immunotherapy.

## 1 Introduction

The significant therapeutic benefits of cancer immunotherapy have positioned it as the leading approach for treating cancer (1, 2). Compared to the use of chemotherapy, the use of the body’s inherent defense mechanisms against cancer as immunotherapy is efficacious and is associated with reduced side effects (3). Given the multifaceted nature of cancer immunotherapy, the role of nursing care cannot be overstated. “Immunotherapy nursing care” refers to the specialized care provided to patients undergoing cancer immunotherapy, including patient education, monitoring immune-related adverse events, symptom management, and psychological support. Nurses play a key role in helping patients understand the treatment process, managing side effects, and offering emotional support. The goal of immunotherapy nursing care is to optimize treatment, minimize side effects, and improve overall patient outcomes. A study showed that in lung cancer patients undergoing immunotherapy, nurses significantly reduced the severity of side effects, such as rashes, fatigue, and gastrointestinal issues, by regularly monitoring side effects and guiding patients on the use of symptomatic treatments. Through early interventions, nurses helped patients maintain treatment continuity and reduced treatment interruptions or hospitalizations caused by side effects (4, 5). Recent research increasingly highlights the critical role of nursing care in the field of cancer immunotherapy. Empirical evidence suggests that through patient education, nurses can enhance patients’ understanding and ability to navigate the complexities of treatment. Additionally, it is imperative for nurses to be adept at managing the potential side effects that may arise, thereby safeguarding patients’ well-being and comfort throughout their treatment journey (1, 6).

Bibliometric analysis is a research method that involves the systematic analysis of published literature to identify trends, patterns, and relationships within a specific field. It typically uses quantitative techniques to examine various aspects of research, such as publication frequency, authorship, citation patterns, and the development of key research topics over time. Previous scholars have conducted massive bibliometric analysis in line with the extant literature and generated knowledge maps in medical research, encompassing cancer immunotherapy, such as breast cancer (7) and bladder cancer (8), as well as specific treatment modalities, such as bispecific antibodies (9). Notably, Yao et al. (10) conducted a comprehensive bibliometric analysis of international cancer immunotherapy, identifying popular research topics and future trends. Despite the growing body of research on cancer immunotherapy nursing, comprehensive literature reviews are lacking. The absence of a systematic overview of the literature hinders the identification of best practices and directions for future research. Dailh et al. (1) conducted a systematic review that emphasized the role of nurses in immunotherapy, particularly in managing adverse events and patient education, but the scope of the study was limited to 79 research articles.

Nevertheless, research on cancer immunotherapy nursing is growing, the existing literature primarily focuses on clinical outcomes and side effect management, with limited exploration of the nursing role in these areas. Specifically, there is a lack of research on how nurses manage immune-related adverse events, provide emotional support, and educate patients during immunotherapy. Additionally, there is a gap in comprehensive, interdisciplinary analysis of global research trends in cancer immunotherapy nursing. By analyzing a large volume of publications, this method helps to reveal key areas of focus, identify influential studies, and provide insights into the evolution of research in this specific field. To clearly outline the research patterns in cancer immunotherapy nursing care in the last two decades and visualize them, this study conducted a bibliometric analysis from 2004 to 2023 and filled the research gap in the literature on cancer immunotherapy nursing care.

## 2 Materials and methods

### 2.1 Study design

This study employed bibliometric analysis, which uses quantitative analysis and statistical methods to describe publication patterns within a specific field, helping to identify research trends and the impact of particular studies or authors.

### 2.2 Inclusion and exclusion criteria

The literature selected for this study adhered to the following inclusion criteria: 1) Full-text publications specifically related to cancer immunotherapy nursing care, including clinical trials, case studies, review articles (including systematic and narrative reviews), educational articles, and studies on patient management strategies; 2) Articles and review manuscripts published in English; 3) Articles published between January 1, 2004, and December 31, 2023. Exclusion criteria included: 1) Publications unrelated to cancer immunotherapy nursing care; 2) Conference abstracts, news articles, and brief communications.

### 2.3 Search strategy

Bibliometric data related to cancer immunotherapy nursing care were retrieved from the Web of Science Core Collection (WoSCC). A comprehensive search was conducted on January 17, 2024, covering the time span from January 1, 2004, to December 31, 2023. The search strategy included combinations of terms related to cancer, nursing care, and immunotherapy. The following search terms were used in the Topic Search (TS): Cancer-related terms: “Neoplasms”, “Tumor”, “Neoplasm”, “Tumors”, “Neoplasia”, “Neoplasias”, “Cancer”, “Cancers”, “Malignant Neoplasm”, “Malignancy”, “Malignancies”, “Malignant Neoplasms”, “Neoplasm, Malignant”, “Neoplasms, Malignant”, “Benign Neoplasms”, “Benign Neoplasm”, “Neoplasms, Benign”, “Neoplasm, Benign”. Nursing-related terms: “Nursing”, “Care”, “Nursing Care”. Immunotherapy: “Immunotherapy”. The final query structure was: TS = (Cancer-related terms) AND TS = (Nursing OR Care OR Nursing Care) AND TS = (Immunotherapy) Filters such as publication years (2004–2023) and document type (articles only) were applied to refine the results. All abbreviations used in this section and the article are clarified at first mention or are listed in a dedicated Abbreviations section.

### 2.4 Data analysis

The research began by extracting and synthesizing relevant publications using Endnote X9. A large sample of peer-reviewed publications over 20 years was analyzed to provide an overview of cancer immunotherapy nursing care research. Visualization tools including GraphPad Prism (version 8.0.2), CiteSpace (version 6.2.4R 64-bit), and VOSviewer (version 1.6.18) were used to explore global research trends, influential players, and citation relationships. GraphPad Prism was employed to analyze publication trends and country-specific contributions over time. Additionally, effect sizes and statistical significance were calculated for studies on nursing interventions in cancer immunotherapy. CiteSpace was used for co-citation analysis to identify key studies and detect emerging research trends. It also helped identify sudden increases in research activity through burst detection. VOSviewer constructed bibliometric networks to map collaborative relationships among authors, institutions, and countries, and identify central research themes. Centrality measures were also used to assess the influence of key players in the field.

## 3 Results

### 3.1 Publication trends

The literature search yielded 4927 results from the WoSCC. Having previously obtained the literature data from the WoSCC, the researchers first excluded studies that were not conducted from 2004 to 2023. After excluding the 137 unqualified publications, the authors excluded studies that belonged to the book chapter, correction, or editorial material category. Aside from the above 145 studies, 119 articles written in languages other than English were also excluded. After applying specific inclusion and exclusion criteria, the sample was refined to 4526 peer-reviewed English articles published between 2004 and 2023, including 2416 articles (84.96%) and 2110 reviews (15.04%). These publications involve 109 countries and regions, 4980 institutions, and 26822 authors.

**Figure 1:**
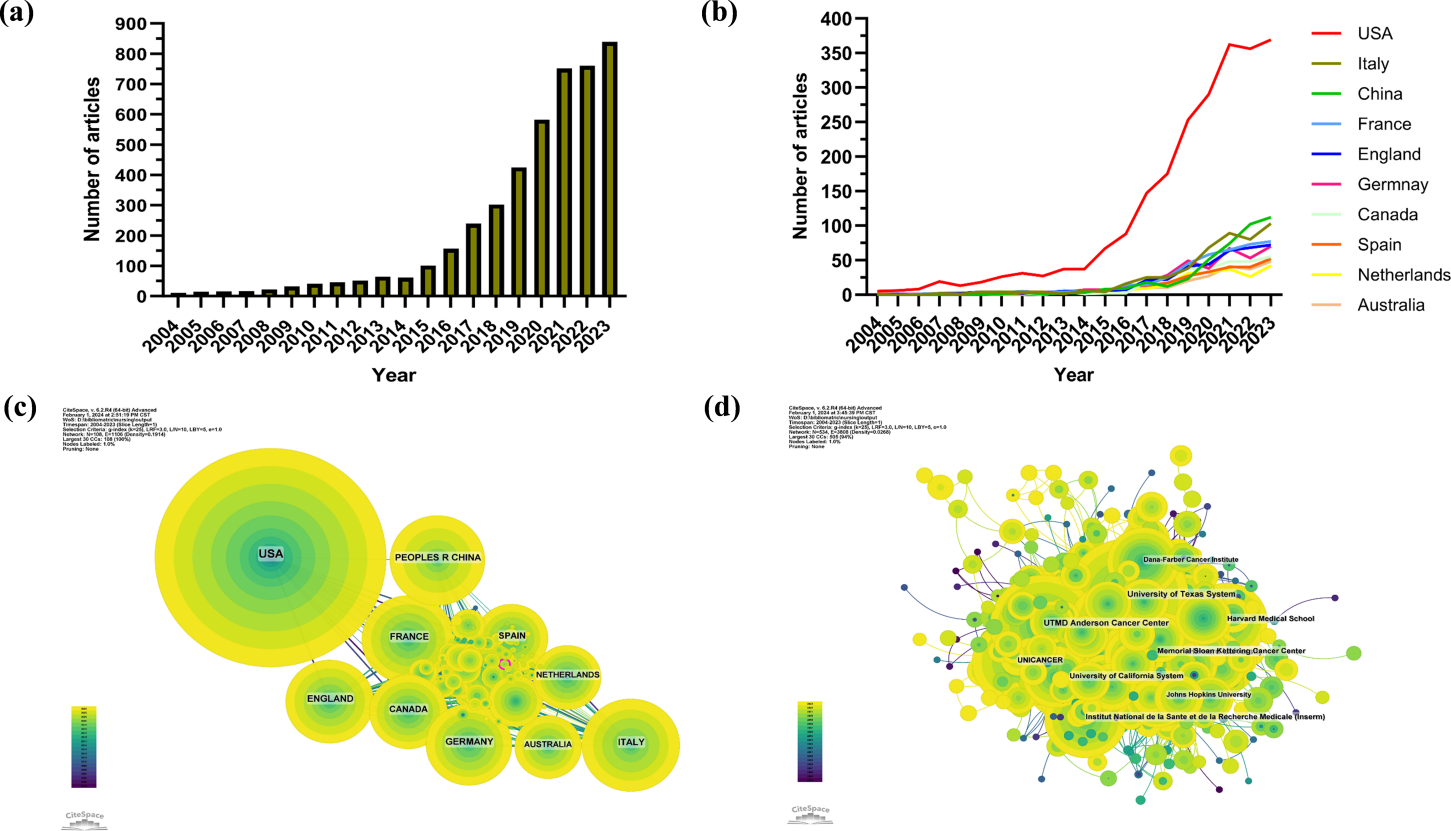
Publication trends and cooperation visualization. **(a)** Number of articles on cancer immunotherapy nursing care. **(b)** Line graph of country publication volume. **(c)** National cooperation network generated by CiteSpace. **(d)** Institutional cooperation network generated by CiteSpace.

The authors described the annual publications on the research topic with GraphPad Prism. **Figure 1a** is a bar chart of the number of publications on cancer immunotherapy nursing care from 2004 to 2023. As **Figure 1a** shows, the number of articles on cancer immunotherapy nursing care is ever increasing annually.

### 3.2 Countries and affiliations

According to the included studies, cancer immunotherapy nursing care applications have been studied in 109 countries and regions. The authors utilized GraphPad Prism to generate **Figure 1b** to display the annual publication volumes of the top 10 countries over the past decades in a line graph. The top 5 countries in this field are the United States, Italy, China, France, and the United Kingdom. **Table 1** reports the top 10 countries publishing works in the research field in terms of their article numbers, centrality, percentage, citations, and citations per article. The United States accounted for 51.36% of the total publications, far exceeding that of other countries. The United States has 87142 publications, thus indicating that the country has strong research capabilities recognized by scholars worldwide. The data from the other nine countries are quite similar across various aspects, suggesting a neck-and-neck situation.

**Table 1:** Top 10 countries according to the search results.

Rank	Country /region	Article numbers	Centrality	Percentage (%)	Citation	Citation per publication
1	USA	2325	0.02	51.36	87142	37.48
2	Italy	475	0.02	10.49	14442	30.40
3	China	419	0.00	9.26	9575	22.85
4	France	401	0.01	8.86	23371	58.28
5	England	373	0.03	8.24	17804	47.73
6	Germany	365	0.03	8.06	17010	46.60
7	Canada	296	0.04	6.54	13921	47.03
8	Spain	255	0.03	5.63	19957	78.26
9	Netherlands	220	0.02	4.86	14322	65.10
10	Australia	215	0.02	4.75	9842	45.78

This study also conducted country and affiliation co-operation analysis via CiteSpace to uncover academic co-operation. It is evident that the United States enjoys the most important position (**Figure 1c**) and is closely connected with France, Canada, Germany, Spain, and other European countries. Although China’s co-operation with other countries is denser than that with the US, it has greater influence than other countries in terms of co-operation strength, with most being European countries. As indicated by **Figure 1d**, institutions that are active in co-operation include UTMD Anderson Cancer Center, Memorial Sloarr Kettering Cancer Center, University of California System, Unicancer, and University of Texas System.

### 3.3 Journal analysis

**Tables 2** and **3** are the top 10 journals with the highest publication volume and the most citations. According to **Table 2**, Cancers with 349 papers, accounting for 7.71% of all 4526 obtained results, is the most prolific journal. *Frontiers in Oncology*, *Journal for Immunotherapy of Cancer*, and *Frontiers in Immunology* are also famous journals on this topic. Among the top ten journals with the most publications, *Clinical Cancer Research* has the highest impact factor (IF) of 11.5. Ninety percent of them are classified as Quartile 1 or Quartile 2 in SCIE journals.

**Table 2:** Top 10 journals in the search results.

Rank	Journal	Article counts	Percentage(4526)	IF	Quartile in category
1	*Cancers*	349	7.71	5.2	Q2
2	*Frontiers in Oncology*	157	3.47	4.7	Q2
3	*Journal for Immunotherapy of Cancer*	104	2.30	10.9	Q1
4	*Frontiers in Immunology*	94	2.08	7.3	Q1
5	*Current Treatment Options in Oncology*	61	1.35	4.3	Q2
6	*Current Oncology*	57	1.26	2.6	Q3
7	*Oncologist*	54	1.19	5.8	Q1
8	*European Journal of Cancer*	53	1.17	8.4	Q1
9	*Clinical Cancer Research*	48	1.06	11.5	Q1
10	*BMC Cancer*	47	1.04	3.8	Q2

**Table 3:** Top 10 cited journals in the search results.

Rank	Cited Journal	Co-Citation	IF (2020)	Quartile in category
1	*Journal of Clinical Oncology*	3634	45.4	Q1
2	*New England Journal of Medicine*	3547	158.5	Q1
3	*The Lancet Oncology*	2674	51.1	Q1
4	*Clinical Cancer Research*	2655	11.5	Q1
5	*Annals of Oncology*	2525	50.5	Q1
6	*The Lancet*	2312	168.9	Q1
7	*Cancer Research*	1846	11.2	Q1
8	*Nature*	1734	64.8	Q1
9	*European Journal of Cancer*	1685	8.4	Q2
10	*JAMA Oncology*	1595	28.4	Q1

**Figure 2:**
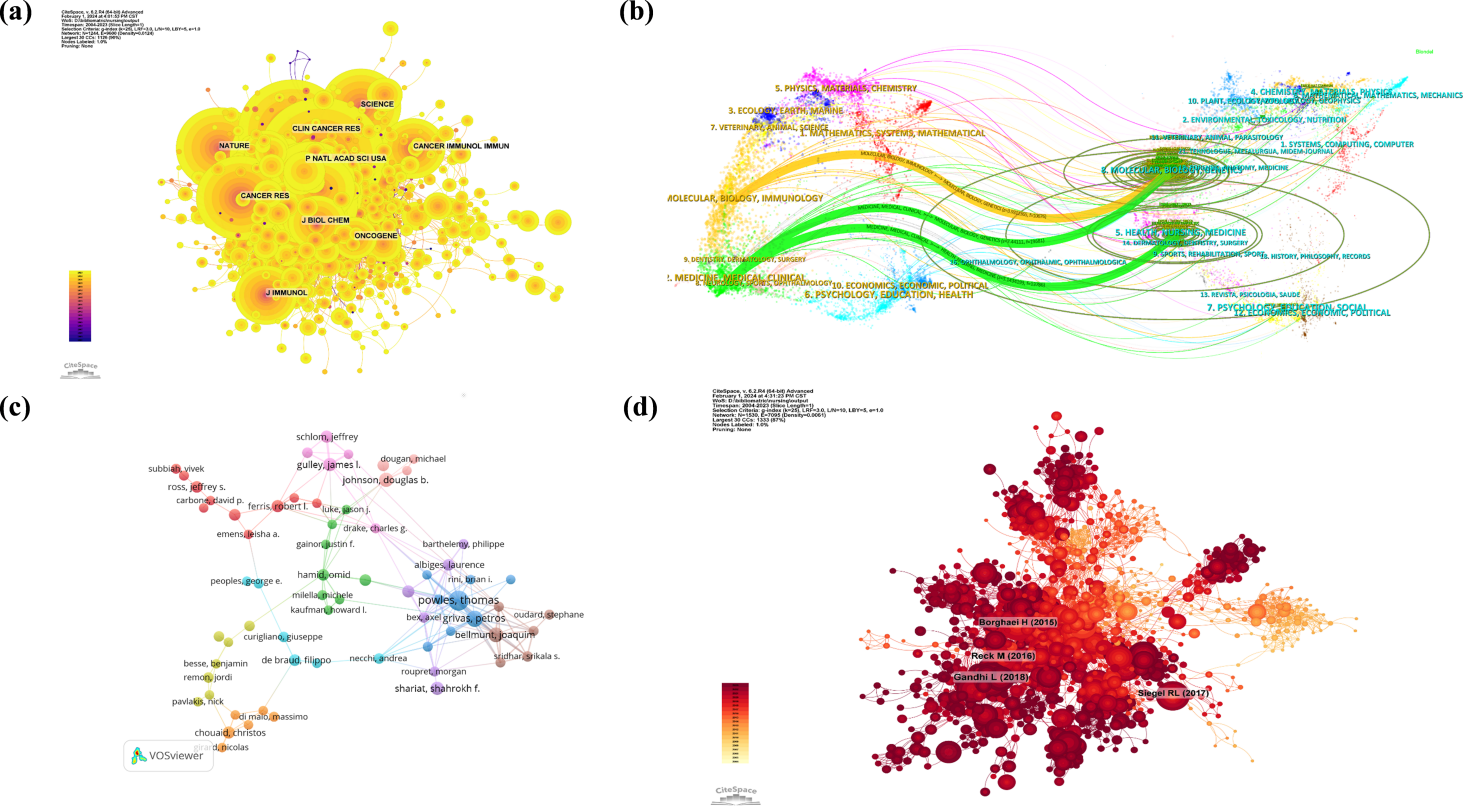
Journal, author and document co-citation visualization. **(a)** Network visualization of journal co-citation analysis (Journal co-citation network generated by CiteSpace. Nodes represent journals, with node size reflecting citation frequency; links indicate co-citation relationships, with thickness representing relationship strength. Colors ranging from blue to red indicate centrality from low to high, with red nodes (e.g., NATURE, SCIENCE) representing core journals). **(b)** A Dual-map overlay of the journal categories (Knowledge flow pathways created by CiteSpace. Left side shows citing journals, right side shows cited journals, with colored lines indicating citation relationships. Thick green lines highlight primary citation paths from medical clinical fields to molecular biology domains, reflecting cross-disciplinary knowledge exchange). **(c)** Co-citation analysis of cited authors (Author influence network generated by VOSviewer. Nodes represent authors, with size reflecting citation frequency; different colors represent distinct research groups; links show co-citation relationships between authors. Powles Thomas and others occupy key node positions, demonstrating their core influence in the field.). **(d)** Network visualization of document co-citation analysis (High-impact document network generated by CiteSpace. Nodes represent documents, with size indicating citation frequency; red nodes (e.g., Borghaei et al., 2015) represent landmark publications; links between nodes show co-citation relationships. Different clusters reflect various research topics such as lung cancer immunotherapy).

The impact factor of a journal is influenced by the frequency of its citations in conjunction with others, indicating whether the journal has had a significant impact on the scientific community. According to **Figure 2a** and **Table 3**, the journal with the most co-citations is the *Journal of Clinical Oncology*, which was cited 3634 times. *The New England Journal of Medicine* and *The Lancet Oncology* were cited 3547 and 2674 times, respectively. Among the top 10 most co-cited journals, *The Lancet* has been cited 2312 times and has the highest IF (IF = 168.9) among the top 10 journals. Among the jointly cited journals, the majority belong to Quartile 1 in SCIE journals.

The theme distribution of academic publications is via a dual overlay map created by CiteSpace, as shown in **Figure 2b**. The colored tracks indicate citation links, with the citing journal on the left side and the cited journal on the right side. The curves represent the citation relationships. According to the visualized results, three main colored citation paths were identified. Research published in journals in the molecular, biology, and immunology fields is mainly cited by research published in journals in the molecular, biology, and genetics fields, while medicine, medical, and clinical studies and health, nursing, and medicine studies are mainly cited by research published in journals in the molecular, biology, and genetics fields.

### 3.4 Authors and document co-citation analysis

Among all scholars who have published relevant literature on cancer immunotherapy nursing care, **Table 4** is among the top 10 authors with the most publications. The top 10 authors published a total of 150 papers. They constituted 3.31% of this field. Thomas Powles has authored the greatest number of research papers, totaling 26. Grivas Petros and Bellmunt Joaquim are following him, with 19 and 6 research articles, respectively. Further analysis revealed that among the top ten authors, eight were from the United States, one was from the United Kingdom, and the other was from France. **Figure 2c** illustrates a visualization of the network between these authors through VOSviewer. **Table 4** shows the top 10 co-cited and most cited authors. A total of 241 authors have been cited more than 50 times, indicating that their research has a high reputation and influence.

**Table 4:** Top 10 authors and co-cited authors in the search results.

Rank	Author	Count	Location	Rank	Co-cited author	Citation
1	Powles Thomas	26	England	1	Reck M.	504
2	Grivas Petros	19	USA	2	Hodi F. S.	495
3	Bellmunt Joaquim	16	USA	3	Brahmer J. R.	447
4	Johnson B. Douglas	15	USA	4	Topalian S. L.	447
5	Gulley L. James	13	USA	5	Siegel R. L.	436
6	Lim Michael	13	USA	6	Robert C.	425
7	Sampson H. John	13	USA	7	Motzer R. J.	412
8	Shariat F. Shahrokh	13	USA	8	Herbst R. S.	372
9	Chouaid Christos	11	France	9	Wolchok J. D.	367
10	Choueiri K. Toni	11	USA	10	Larkin J.	348

Taking one year as the time period and the time range from 2004 to 2023, the co-citation reference network can be seen in **Figure 2d** with 1530 nodes and 7095 links. According to the top 10 most co-cited articles in **Figure 2d**, the article titled “Pembrolizumab plus Chemotherapy in Metastatic Non-Small Cell Lung Cancer” in *The New England Journal of Medicine* (IF=158.5) is the most co-cited reference (11). Gandhi L. is the first author of this article for advanced NSCLC patients lacking targetable mutations.

**Figure 3:**
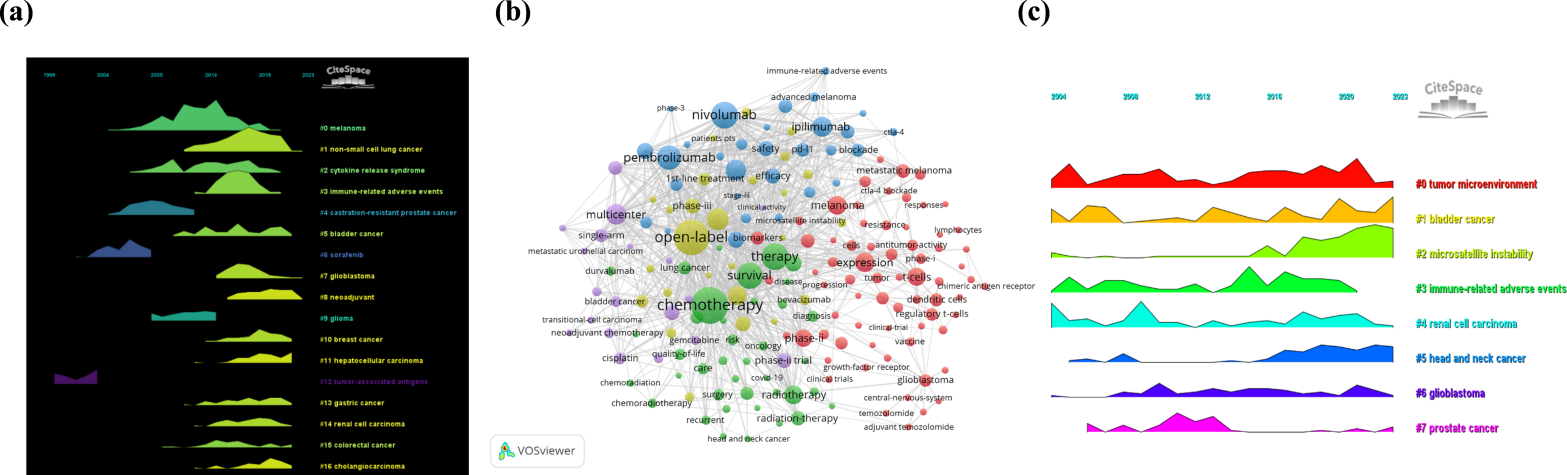
Chronological clustering visualization of document and keywords. **(a)** Chronological clustering visualization of document co-citation analysis by CiteSpace (Colored streams represent research clusters (e.g., #0 melanoma, #1 non-small cell lung cancer); horizontal axis spans 2004-2020; stream width variations reflect changes in research intensity. Shows the shift from early hotspots (#12 tumor-associated antigen) to recent focuses (#3 immune-related adverse events)). **(b)** Co-occurrence of heated-debated keywords network visualization on the research topic via VOSviewer (Nodes represent keywords, with size reflecting occurrence frequency; five colors distinguish concept groups: expression and T cells (red), chemotherapy and survival (green), PD-L1 and adverse events (blue), open-label and targeted therapy (yellow), multicenter clinical trials (purple); links indicate keyword co-occurrence). **(c)** Chronological clustering visualization of keywords analysis through CiteSpace (Horizontal axis represents 2004–2022 timeline; colored curves represent different research topics (e.g., #0 tumor microenvironment, #2 microsatellite instability); curve height variations reflect changes in topic intensity).

The authors then performed co-citation reference clustering and temporal clustering analysis, as shown in **Figure 3a**. Cluster 12, a tumor-associated antigen, was identified as an early research hotspot. Cluster 4, castration-resistant prostate cancer; cluster 6, sorafenib; and cluster 8, glioma, are mid-term research hotspots. Others, namely, melanoma (cluster 0), non-small cell lung cancer (cluster 1), cytokine release syndrome (cluster 2), immune-related adverse events (cluster 3), bladder cancer (cluster 5), glioblastoma (cluster 7), neoadjuvant therapy (cluster 8), breast cancer (cluster 10), hepatocellular carcinoma (cluster 11), gastric cancer (cluster 13), renal cell carcinoma (cluster 14), colorectal cancer (cluster 15) and cholangiocarcinoma (cluster 16), are hot topics and trends in this field.

### 3.5 Clustering analysis with keywords

Keywords analysis allows researchers to quickly grasp the situation and development direction of an academia. **Figure 3b** shows the visualization of the occurrence of the keywords produced by VOSviewer. According to **Figure 3b**, the most popular keyword is chemotherapy (occurrence = 854). The following items were used: open-label (occurrence = 804), nivolumab (occurrence = 511) and survival (occurrence = 483). The authors removed useless keywords and constructed a network containing 183 keywords that appeared at least 12 times, resulting in a total of 5 different clusters.

Cluster 1 (red) included 69 keywords related to expression, T cells, dendritic cells, vaccines, melanoma, regulatory T cells, lymphocytes, resistance, clinical trials, glioblastoma, the central nervous system, long-term survival, immunosuppression, lung cancer, progression, microsatellite instability, combination, and metastatic melanoma. Cluster 2 (green) had 39 keywords, including chemotherapy, therapy, survival, radiotherapy, head and neck cancer, care, surgery, quality of life, recurrence, outpatient, palliative care, COVID-19, and impact. Cluster 3 (blue) contains 30 keywords, such as pd-l1, nivolumab, toxicity, adverse events, anti-PD-1, stage III, immune-related adverse events, safety, efficacy, cost effectiveness, biomarker, and tumor mutational burden. There are 26 keywords in Cluster 4 (yellow), including open-label, placebo, 1st-line therapy, targeted therapy, supportive care, patients pts, sunitinib, interferon alpha, tyrosine kinase inhibitor, randomized trial, and randomized phase-ii. Cluster 5 (purple) consisted of 19 keywords, namely, multicenter, single-arm, transitional cell carcinoma, neoadjuvant chemotherapy, cisplatin, radical cystectomy, gemcitabine, paclitaxel, carboplatin, atezolizumab, and clinical activity. The authors then created a volcano map through CiteSpace to visually display the changes in research hotspots over time, as shown in **Figure 3c**.

### 3.6 Burst detection analysis with references and keywords

**Figure 4:**
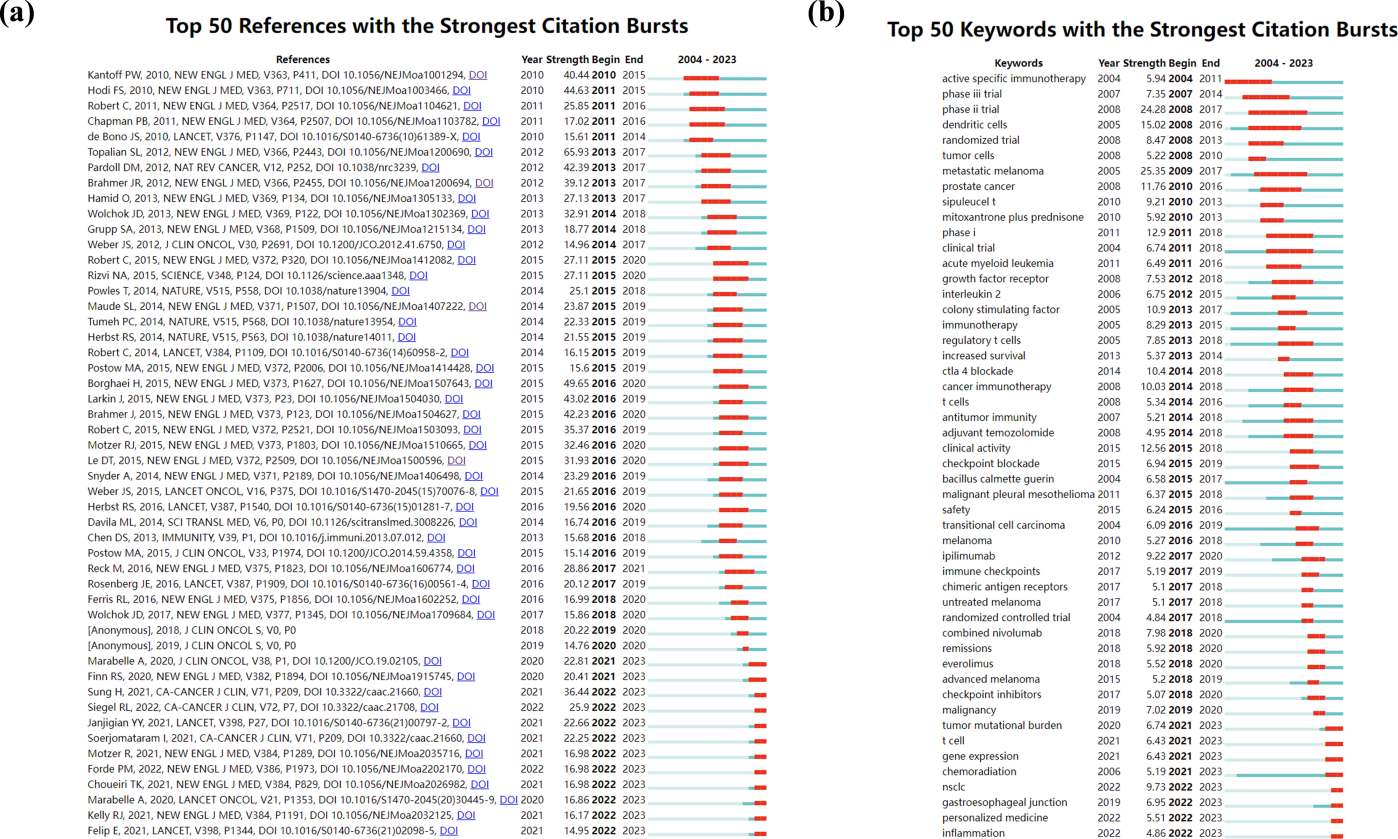
Top 50 references **(a)** and keywords **(b)** with the strongest citation bursts.

Using CiteSpace, the researchers identified the 50 most reliable citation bursts in the field of cancer immunotherapy care (**Figure 4a**). CiteSpace provides a burst detection function to detect significant changes in citation frequency during a certain period, aiming to identify the emergence or decline of a specific topic or keyword (12, 13). In the burst plot, the red line represents the specific duration phases during which the keyword becomes a hot topic in academic research, light blue indicates nodes that have not yet appeared, and dark blue indicates nodes that have begun to appear. The functionality of burst detection has been applied in bibliometric analysis in the field of medicine (7, 14, 15).

The reference with the highest citation rate (49.65%) was “Nivolumab versus Docetaxel in Advanced Nonsquamous Non-Small Cell Lung Cancer”, which was published in the *New England Journal of Medicine*. The first author of this article is Hossein Borghaei, D. O. Nivolumab is a fully human IgG4 programmed death 1 (PD-1) immune checkpoint inhibitor antibody that can disrupt PD-1-mediated signaling, thereby restoring antitumor immunity (16). Fifty of the references were published between 2004 and 2023, which suggests that these papers have been cited frequently over the last 20 years. Twelve of these papers are currently at peak citation, implying that cancer immunotherapy nursing research will continue to be of interest in the future.

Among the 786 strongest burst keywords in this field, the authors focused on the 50 keywords with the strongest citation bursts (**Figure 4b**). These keywords represent the current research hotspots and possible future research directions (17). Research trends in the field of cancer immunotherapy nursing care were also shown at the beginning and end of the study period. From 2004 to 2008, active specific immunotherapy, phase III trials, phase II trials, dendritic cell trials, randomized trials, and tumor cell studies were popular among researchers, with the latest burst having concluded at least five years ago. The burst plots of these keywords, such as “metastatic melanoma”, “prostate cancer”, “clinical trial”, and “regulatory T cells”, gradually turned red between 2009 and 2013. The most recent burst also ended at least five years ago. According to **Figure 4**, ipilimumab, safety, clinical activity, immune checkpoints, and untreated melanoma were research hotspots, with relatively short burst durations. The figure also clearly provides the authors with the hotspots in cancer immunotherapy nursing care in the past five years. Malignancy, tumor mutational burden, gene expression, chemoradiation, NSCLC, gastroesophageal junction, personalized medicine, and inflammation are gaining popularity among scholars, and currently, they are still under heated discussion.

## 4 Discussion

In modern healthcare systems, nurses enjoy high positions in the care of patients undergoing immunotherapy (3). Zhang and Lu (18) discussed follow-up care involving immune checkpoint inhibitors and acknowledged the importance of nursing care. Compared to other members of the healthcare team, they need to not only possess professional nursing skills but also be familiar with relevant knowledge of immunotherapy to provide comprehensive care services to patients more effectively (19). It is widely acknowledged that all scopes of practice are underpinned by ethical nursing principles to ensure accountability for all aspects of patient care (20). Previous scholars have conducted numerous bibliometric analysis based on the extant literature and generated knowledge maps in the field of medicine (6, 21). It is also applied in specific cancer immunotherapies. Y. Liu once conducted a bibliometric analysis on the global landscape of lung cancer immunotherapy (22). The top papers, top journals, and research hotspots on this topic were also identified. Zhong performed a bibliometric analysis focusing on immunotherapy for prostate cancer, summarizing the prevailing trends using knowledge mapping methods (23). Y. Liu discussed the research trends and meaningful clinical experiments in anti-PD1/PDL1 cancer immunotherapy through bibliometric analysis of the literature (24). A bibliometric analysis of bladder cancer immunotherapy was performed via VOSviewer (8). Cooperative relationships were identified through citation, co-citation analysis, and co-authorship in the field of bladder cancer from 2000 to 2022. Nevertheless, there is no bibliometric analysis on cancer immunotherapy nursing care, which summarizes its global research trends over the last two decades and provides future implications.

To clearly outline the research patterns in cancer immunotherapy nursing care in the last two decades and visualize them, this study conducted a bibliometric analysis of cancer immunotherapy nursing care from 2004 to 2023. Researchers have found that since 2004, the number of publications per year has gradually increased. The period was divided into three phases. The first stage was slow growth from 2004 to 2011, with fewer than 50 papers published annually, indicating that limited attention has been given to this field. The second period can be regarded as a gradual increase in publications from 2012 to 2016, suggesting increasing interest from researchers. In the third stage, the number of papers published increased rapidly after 2017, reaching a peak in 2023, indicating that the field has received widespread attention since 2017.

As presented in **Table 1**, there are top ten reproductive countries or regions ranked by publication volume. This study revealed that the United States has the most citations, far surpassing all other countries and regions. Its citation-to-publication ratio (37.48%) ranks 8th among all countries and regions. Italy is the second-highest country in terms of publication volume, with 14442 citations, ranking 6th. However, its citation-to-publication ratio (30.40%) is relatively low, indicating generally lower article quality. The collaboration network indicates close collaboration between two high-producing countries, the United States and Italy. The United States collaborates closely with other countries, including China, France, and the United Kingdom, while Italy’s collaboration is closer to countries such as Australia, the Netherlands, and Germany. The United States has not only a large publication volume but also a high citation frequency, indicating its leading position in the field. In recent years, countries such as the United Kingdom and France have experienced rapid increases in publication volume, possibly due to collaboration with the United States.

A total of 4980 institutions systematically published articles on cancer immunotherapy nursing care. Among the top ten institutions ranked by publication volume, eight are from the United States, and two are from France. The University of Texas System has the highest number of publications (359 papers, cited 11791 times, averaging 32.84 citations per paper). Harvard University (293 papers, cited 15704 times, averaging 53.60 citations per paper) ranks second, followed by UTMD Anderson Cancer Center (287 papers, cited 9619 times, averaging 33.52 citations per paper). Further analysis revealed that both domestic and foreign agencies tended to work with their domestic counterparts. Therefore, we advocate strengthening co-operation between domestic and foreign institutions to overcome academic barriers.

Through journal analysis, the researchers found that *Cancers* published the most articles in the field of cancer immunotherapy nursing care, followed by *Frontiers in Oncology* and the *Journal for Immunotherapy of Cancer*. The *Journal of Clinical Oncology* was identified as the most co-cited journal in the following analysis. It is aligned with the current study, and the author did find several meaningful studies, which were all published in these journals. Kamat et al. (25) focused on the use of immunotherapy, particularly the intravesical Bacillus Calmette-Guerin (BCG), as the standard treatment for non-muscle-invasive bladder cancer (NMIBC). Londoño et al. (26) discussed the management of side effects from checkpoint inhibitor drugs in cancer treatment, emphasizing the need for a multidisciplinary approach and early detection of adverse events to ensure optimal patient care. These articles have received significant citations, demonstrating the influence of these journals in the academic community.

The current study utilized both CiteSpace and VOSviewer to explore the prolific authors and co-authors in the research field. Powles Thomas and Martin Reck were deemed the most reproductive authors and co-authors. Powles Thomas and his colleagues have reported on numerous clinical trials advancing the research theme of metastatic urothelial carcinoma. Powles et al. (27) compared the efficacy and safety of atezolizumab versus chemotherapy in patients with platinum-refractory metastatic urothelial carcinoma overexpressing PD-L1 (IC2/3) and reported that atezolizumab had a similar overall survival but a more favorable safety profile. Long-term follow-up over 5 years demonstrated that pembrolizumab continues to exhibit durable efficacy without new safety signals in patients with platinum-resistant metastatic urothelial carcinoma and as a first-line therapy in cisplatin-ineligible patients (28). Apart from the improvement of metastatic urothelial carcinoma, Choueiri et al. (29) also presented analysis of health-related quality of life in patients enrolled in the KEYNOTE-564 trial, demonstrating that adjuvant treatment with pembrolizumab did not lead to deterioration of HRQoL, supporting its use following nephrectomy for renal cell carcinoma.

Researchers would like to further discuss the most co-cited articles. The first-line therapy is platinum-based chemotherapy. In patients with a programmed death-ligand 1 (PD-L1) tumor proportion score of 50% or more, pembrolizumab has replaced cytotoxic chemotherapy as the first-line treatment. In a phase II trial, the addition of pembrolizumab to chemotherapy significantly improved the response rate and prolonged progression-free survival (PFS) compared with chemotherapy alone. In this double-blind phase 3 trial, 616 patients (ratio 2:1) were randomly assigned. The patients received pemetrexed and platinum plus 200 mg pembrolizumab every 3 weeks or placebo for 4 cycles and then received pembrolizumab or placebo for up to 35 cycles plus pemetrexed. Trecet maintenance treatment. Patients in the placebo combination therapy group could crossover to pembrolizumab monotherapy if their disease progressed. The primary endpoints were overall survival and PFS, which were assessed by blinded independent central radiologists.

After a median follow-up of 10.5 months, the estimated 12-month overall survival rate was 69.2% (95% confidence interval [CI] [64.1, 73.8]) in the pembrolizumab combination group and 49.4% (95% CI [42.1, 56.2]) in the placebo combination group). Hazard ratio of death was 0.49 (95% CI [0.38, 0.64]; p < 0.001). Overall survival improved across all PD-L1 categories evaluated. The median PFS was 8.8 months (95% CI [7.6, 9.2]) in the pembrolizumab combination group and 4.9 months (95% CI [4.7, 5.5]) in the placebo combination group (hazard ratio for disease progression or death, 0.52; 95% CI [0.43, 0.64]; p < 0.001). Grade 3 or higher adverse events occurred in 67.2% of patients in the pembrolizumab combination group and 65.8% of patients in the placebo combination group. This article considers the addition of pembrolizumab to standard chemotherapy with pemetrexed and a platinum-based drug for previously untreated patients with metastatic nonsquamous NSCLC without epidermal growth factor receptor (EGFR) or ALK mutations. Overall survival (OS) and PFS were significantly longer in these patients than in those treated with chemotherapy alone.

The clustering analysis with keywords was conducted by CiteSpace and VOSviewer, each of which generated a network and chronological visualization. This finding indicates that chemical and immunotherapeutic nursing care are often compared and discussed in academic articles as two different approaches to cancer treatment (4, 5, 30, 31). This study also identified other clusters comprising the tumor microenvironment, immune-related adverse events, microsatellite instability, and bladder cancer.

The first two clusters were highly important before 2020 but ceased to become research hotspots thereafter. Postow et al. (32) once published academic papers reporting the association between immune-related adverse events and immune checkpoint blockade and have become highly cited. Recommendations for specific organ system-based toxicity diagnosis and management were made according to the American Society of Clinical Oncology Clinical Practice Guidelines (33). The latter two clusters have been research hotspots in the past three years, and their research momentum is expected to remain strong in the future. Vartolomei et al. (34) examined the prevalence of depression, anxiety, and suicide among bladder cancer patients, highlighting the need for improved identification and management of psychological distress in this population. Tang et al. (35) conducted a meta-analysis to evaluate the effectiveness and safety of immune checkpoint inhibitors in treating microsatellite instability-high colorectal cancer through a comprehensive pooled analysis of clinical trial data.

The authors performed burst detection of references and keywords in CiteSpace to chronologically present the research trends. In this randomized, open-label, international phase III study (16), the reference with the highest citation rate, researchers assigned patients with nonsquamous non-small cell lung cancer who progressed during or after platinum-based two-agent chemotherapy to receive nivolumab at a dose of 3 mg per kilogram of body weight every two weeks or docetaxel at a dose of 75 mg per square meter of body surface area every three weeks. The primary endpoint was overall survival. Patients treated with nivolumab had longer overall survival than those treated with docetaxel. The median overall survival was 12.2 months (95% CI [9.7, 15.0]) for the 292 patients in the nivolumab group and 9.4 months (95% CI [8.1, 10.7]) for the 290 patients in the docetaxel group (hazard ratio for death 0.73; 96% CI [0.59, 0.89]; p = 0.002). At 1 year, the overall survival rate was 51% (95% CI [45, 56]) for nivolumab and 39% (95% CI [33, 45]) for docetaxel.

After further follow-up, the 18-month overall survival rate was 39% (95% CI [34, 45]) for nivolumab and 23% (95% CI [19, 28]) for docetaxel. The response rate to nivolumab was 19%, whereas that to docetaxel was 12% (p = 0.02). Although progression-free survival was not superior to that of patients treated with docetaxel plus nivolumab (median 2.3 and 4.2 months, respectively), the 1-year progression-free survival rate was greater with nivolumab than with docetaxel (19% and 8%, respectively). In subgroups defined by prespecified levels of tumor membrane expression of PD-1 ligands (≥1%, ≥5%, and ≥10%), nivolumab was more efficacious than docetaxel at all endpoints. Ten percent of patients in the nivolumab arm reported grade 3 or 4 treatment-related adverse events, compared with 54% of patients in the docetaxel arm. Overall survival was longer with nivolumab than with docetaxel in patients with advanced nonsquamous NSCLC whose disease worsened during or after platinum-based chemotherapy. Through the burst detection of references, the authors determined that non-small cell lung cancer has indeed been a very hot research topic in the past decade, garnering numerous researchers’ studies and citations (16, 36–38), and the topic’s popularity shows no signs of waning. This finding is consistent with the results of burst detection on keywords.

The quality of this study is evident through its robust search strategy, comprehensive sample size, use of multiple bibliometric tools, global perspective, and well-defined inclusion criteria. First, the search strategy employed a comprehensive list of cancer-, tumor-, and neoplasm-related terminologies, ensuring wide coverage of publications. Second, the analysis included a large sample size of 4526 publications, which can provide a comprehensive overview of the research landscape in cancer immunotherapy nursing care. Third, the use of multiple bibliometric software tools enhanced the depth of analysis, enabling thoroughly visualized outcomes. Fourth, the study included publications from 109 countries, demonstrating a global perspective on cancer immunotherapy nursing care research. Finally, the inclusion criteria were clearly defined, limiting the analysis to peer-reviewed articles published in English from 2004 to 2023, ensuring the quality of the publications analyzed.

Several limitations should be acknowledged. First, this bibliometric analysis focused merely on the research trend from 2004 to 2023. Earlier papers failed to be involved. Second, studies that were written in languages other than English were not included, probably resulting in publication bias and thus rendering the whole analysis less comprehensive. Third, because of the limited document readability of VOSviewer, the researchers selected only publications from the WoSCC. Future studies are expected to expand the sample range of databases to systematically investigate the advantages of nursing care, providing valuable insights for clinical practice.

## 5 Conclusion

To the authors’ knowledge, the current study is the first bibliometric analysis of cancer immunotherapy nursing care in a relatively academic and comprehensive way. With the assistance of the visualization software programs GraphPad Prism, VOSviewer, and CiteSpace, the authors bibliometrically presented and analyzed the landscape of cancer immunotherapy nursing care from 2004 to 2023. The results indicated that 1) the publication trend of the topic is ever increasing every year; 2) the United States and its University of Texas System are the most prolific country and institution, respectively; 3) the majority of the top ten journals with the most publications are classified as Quartile 1 or Quartile 2 of SCIE journals with high impact factors; 4) Powles Thomas and Martin Reck have authored or co-authored the greatest number of research papers; and 5) keywords such as “melanoma”, “non-small cell lung cancer”, “cytokine release syndrome”, and “immune-related adverse events” were identified as hot topics. The researchers also summarized the hotspots in chronological order and further discussed the co-cited relationships among references. This study is conducive to helping future scholars to comprehensively and quantitively determine the current research trends in cancer immunotherapy nursing care.

## Data Availability

The raw data supporting the conclusions of this article will be made available by the authors, without undue reservation.
